# Adenosine Signaling in Autoimmune Disorders

**DOI:** 10.3390/ph13090260

**Published:** 2020-09-22

**Authors:** Giulia Magni, Stefania Ceruti

**Affiliations:** Laboratory of Cellular and Molecular Pharmacology of Purinergic Transmission, Department of Pharmacological and Biomolecular Sciences, Università degli Studi di Milano, 20133 Milan, Italy; giulia.magni@unimi.it

**Keywords:** autoimmunity, adenosine receptors, CD73, CD39, multiple sclerosis, rheumatoid arthritis, psoriasis, methotrexate, IB-MECA

## Abstract

The molecular components of the purinergic system (i.e., receptors, metabolizing enzymes and membrane transporters) are widely expressed in the cells of the immune system. Additionally, high concentrations of adenosine are generated from the hydrolysis of ATP in any “danger” condition, when oxygen and energy availability dramatically drops. Therefore, adenosine acts as a retaliatory metabolite to counteract the nucleotide-mediated boost of the immune reaction. Based on this observation, it can be foreseen that the recruitment with selective agonists of the receptors involved in the immunomodulatory effect of adenosine might represent an innovative anti-inflammatory approach with potential exploitation in autoimmune disorders. Quite surprisingly, pro-inflammatory activity exerted by some adenosine receptors has been also identified, thus paving the way for the hypothesis that at least some autoimmune disorders may be caused by a derailment of adenosine signaling. In this review article, we provide a general overview of the roles played by adenosine on immune cells with a specific focus on the development of adenosine-based therapies for autoimmune disorders, as demonstrated by the exciting data from concluded and ongoing clinical trials.

## 1. The Immunomodulatory Role of Adenosine

### 1.1. The Equilibrium between ATP and Adenosine Signaling in Inflammation and Traumatic Conditions

The adenine nucleotide ATP is now recognized as one of the fundamental biochemical signals controlling cell-to-cell communication within all tissues, including the immune system. Under physiological conditions, the concerted actions of several metabolizing enzymes (i.e., CD73 and CD39, see also below) maintain the extracellular concentrations of ATP within the nanomolar range. However, concentrations can raise up to the high micromolar range following cell lysis, traumatic events, anoxic conditions, or during inflammatory processes, all leading to either the massive release of the intracellular nucleotide pool or to reduced oxygen supply. In particular, ATP behaves as a damage-associated molecular pattern (DAMP) which triggers and promotes inflammatory processes through the recruitment of its ionotropic P2X and metabotropic P2Y receptors. Conversely, the end-product of ATP catabolism, adenosine (Ado), behaves as a “retaliatory metabolite” which is endowed with specific anti-inflammatory and immunomodulatory properties, thus allowing the timely switching off of body reactions to harmful events [1]. This is achieved through the enrolment of the four Ado receptor subtypes, namely the A_1_, A_2A_, A_2B_ and A_3_ Ado receptors (ARs; see below for further details). A_1_ARs and A_3_ARs are coupled to phospholipase C (PLC) and inositol 1,4,5-trisphosphate (IP3) formation, whereas the A_2A_ARs and A_2B_ARs stimulate adenylyl cyclase through G_s_, but different signaling pathways can be recruited in specific tissues and organs (see below) [2].

Modifications in the fine-tuning of the inflammatory responses lead to excessive and prolonged inflammation and, as a consequence, to chronic inflammatory diseases. Additionally, the altered and prolonged immune recognition of self-proteins as non-self antigens leads to the development of autoimmune disorders, and a dysregulation of Ado immunomodulatory signaling pathways can contribute to this process. In this review article, we have summarized the vast literature that has been published on the immunomodulatory role of Ado under both physiological and pathological conditions, along with data demonstrating the involvement of Ado signaling in the mechanisms of actions of known anti-inflammatory and immunomodulatory drugs. Finally, the most relevant encouraging data on the possible future development of specific Ado-mediated pharmacological approaches to autoimmune disorders are discussed and commented.

### 1.2. Adenosine Receptors and Immune Cells

The immune system, composed by dedicated organs, cells and signaling molecules, has the pivotal role of maintaining the homeostasis of the organism, also through the protection from harmful substances, germs, and cellular modifications that could potentially cause damage. The immune response could be innate (non-specific) or adaptive (specific). They are closely interconnected and work together whenever the immune system should be recruited. Specifically, the innate immune system provides the first general defense against non-self antigens through a very quick and non-specific response. Innate immunity is composed by the skin and mucous membranes as a first defense barrier, followed by the recruitment of immune cells including natural killer (NK) cells, mast cells, and phagocytes (i.e., neutrophils, macrophages, and dendritic cells). Overall, through the production of chemical mediators (e.g., cytokines and chemokines) and activation of the complement cascade, innate immunity promotes inflammation and clearance of antibody complexes or dead cells, identification and removal of non-self molecules from organs, tissues, blood and lymph by specialized cells, and activation of the adaptive immune system thanks to a process known as antigen presentation. The adaptive immune system includes both antibody and cell-mediated responses, carried out by two classes of lymphocytes (B cells and T cells, respectively). It is also known as “acquired” immune response, and it is responsible for both the immediate and the long-lasting reactions to specific antigens. Unlike the innate immune system, the adaptive immune system is highly specific to a particular pathogen through the recognition of non-self antigens during the process of antigen presentation, the generation of responses tailored to eliminate specific pathogens or pathogen-infected cells, and the development of immunological memory, in which pathogens are “remembered” through memory B and T cells and will be then more easily eliminated in the case of subsequent recognition [3].

The immunomodulatory role of Ado has been first described back to the early 70s, with the discovery of its role in the development and function of different immune cell populations [4], in parallel with the first report linking a defective Ado metabolism (Ado deaminase, ADA, deficiency) with the onset of severe combined immunodeficiency (ADA-SCID) [5].

From then on, a number of studies progressively addressed and confirmed a role for Ado in immune cell maturation, proliferation and functions, and supported the involvement of Ado signaling in the pharmacological effects of several immunomodulatory drugs.

#### 1.2.1. Innate Immunity

**Figure 1:**
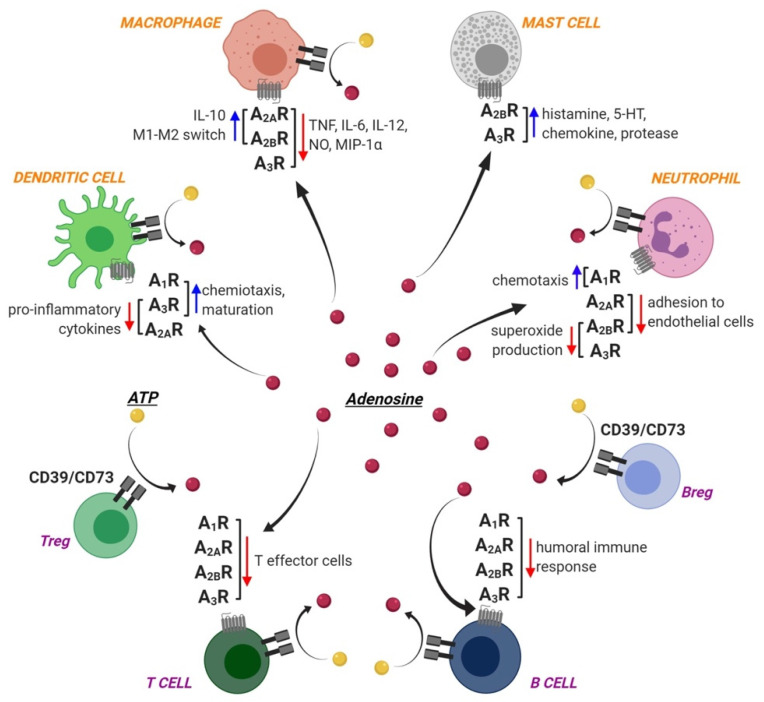
Schematic representation of Ado receptor expression and functions on innate immunity (orange text) and acquired immunity (purple text) cells. Red and blue arrows represent functions that are reduced and enhanced, respectively, by Ado binding to the different receptor subtypes. ATP-metabolizing enzymes CD73 and CD39 are also shown, which cooperate in the generation of extracellular Ado (red dots) through ATP (yellow dots) hydrolysis, thus shifting the balance towards an immunosuppressive environment. Both CD39 and CD73 are highly expressed by T_regs_ and B_regs_, which act as functional regulators for anti-inflammatory and immunosuppressive action during inflammation/infection. See text for details. Created with BioRender.com.

Ado receptors are expressed by different cells of the innate immunity, thus contributing to their function, as comprehensively summarized in Reference [2], and schematically illustrated in Figure 1.

Ado can modulate several functions of dendritic cells, which act as specific antigen-presenting cells driving the first phase of adaptive immunity activation. In particular, chemotaxis of immature dendritic cells is modulated by A_1_ARs and A_3_ARs, while mature dendritic cells mainly express A_2A_ARs and A_3_ARs, which reduce the release of pro-inflammatory cytokines [6]. Conversely, A_2B_ARs drive dendritic cell differentiation towards a pro-angiogenic and pro-inflammatory phenotype [7]. Additionally, the pro-inflammatory effect of A_2B_ARs is also related to the formation of a molecular complex with the ecto-ADA enzyme. Indeed, ADA is released into the extracellular space by immune cells, and it anchors to membrane proteins such as the T cell-activation molecule CD26 and ARs. Thus, on one hand, the enzymatic activity of ADA reduces Ado levels and contributes to immune regulation, but on the other hand, ADA delivers signals to T lymphocytes by acting as a bridge between A_2B_ARs on dendritic cells and CD26 on T-cells. Thus, ecto-ADA acts as a costimulatory molecule through its catalytic-independent activity, and in turn promotes TNF-α and IFN-γ production by T-cells [8].

Mast cells belong to the innate immunity as well, and are activated by direct binding of pathogens or of pathogen-associated molecular patterns (PAMPs) to their surface, through Toll-like receptors (TLRs) or receptors for complement [9]. This results in the release of inflammatory mediators, which contributes to eliminating pathogens also through the regulation of other cells contributing to the immune reaction, including dendritic cells, macrophages, T and B lymphocytes, fibroblasts, eosinophils, endothelial and epithelial cells [9]. Mast cells express A_2B_ARs and A_3_ARs, and in vitro studies on murine mast cells showed that activation of these receptor subtypes stimulates cell degranulation, which in turn induces histamine, serotonin, chemokine and protease release [10], thus contributing to the modulation of immune cell functions. Studies on human mast cells are a little more controversial, since it has been reported that A_2B_ARs are mostly involved in promoting cell degranulation, while A_3_ARs mediate anti-inflammatory effects. Moreover, the pharmacological stimulation of A_3_ARs potentiates human lung but not skin mast cell degranulation, suggesting a site-specific role for A_3_ARs in the immune response [11].

Ado signaling is also involved in monocyte and macrophage maturation and function with a quite complex outcome: quiescent monocytes are characterized by a low expression of A_1_ARs, A_2A_ARs and A_3_ARs, while their expression levels increase during differentiation into macrophages, under the regulation of pro-inflammatory cytokines and of Ado itself [2]. Macrophages also express A_2B_ARs, and evidence shows that, following TLR stimulation, they upregulate this receptor subtype to enhance their sensitivity to immunosuppressive extracellular Ado. By contrast, IFN-γ inhibits A_2B_AR expression, thus reducing macrophage sensitivity to Ado and preventing cell transition towards an immunoregulatory phenotype [12]. Thus, different pro-inflammatory stimuli show opposite outcomes on A_2B_ARs, and in the end on the immunomodulatory actions of Ado. Additionally, A_2A_AR, A_2B_AR, and A_3_AR activation reduces the macrophage release of several pro-inflammatory mediators, including TNF-α, IL-6, IL-12, nitric oxide (NO), and macrophage inflammatory protein (MIP)-1α [2]. In parallel, extracellular Ado promotes the release of the anti-inflammatory cytokine IL-10 by monocytes and macrophages through A_2A_ARs and A_2B_ARs [2]. Finally, it has been demonstrated that A_2A_AR and A_2B_AR activation promotes the phenotypic switch of macrophages from pro-inflammatory M1 to anti-inflammatory M2 phenotype [13]. Similarly to other cells of both the innate and acquired immunity (e.g., dendritic cells, granulocytes, B-cells, and T-cell subsets), macrophages express two key enzymes in purine nucleotide catabolism (Figure 1): ecto-nucleoside triphosphate diphosphohydrolase 1 (CD39 or E-NTPDase1), which catalyzes the sequential degradation of ATP to ADP and AMP, and ecto-5′-nucleotidase (CD73), which dephosphorylates AMP. It has been reported that pro-inflammatory M1 macrophages display a decreased expression and activity of both enzymes, leading to reduced ATP degradation. By contrast, anti-inflammatory M2 macrophages showed an increased expression and activity of CD39 and CD73, bearing to an increase in the conversion of ATP into Ado. Therefore, anti-inflammatory M2 macrophages generate an Ado-rich environment, which in turn contributes to sustaining the anti-inflammatory and tissue remodeling activities of these cells [14].

Ado also regulates the activation of neutrophils, the most abundant leukocytes acting as a first line of defense of the innate immunity. Neutrophils also express both CD39 and CD73 enzymes and represent an abundant source of Ado, which in turn regulates their activation under both normal and inflammatory conditions [15], acting on its four receptors that are all expressed by this cell population [16]. In particular, A_1_ARs promote neutrophil chemotaxis, while A_2A_AR and A_2B_AR activation inhibit neutrophils’ adhesion to endothelial cells [2]. A_2A_ARs were also shown to reduce the release of IL-8, which promotes leukocyte chemoattraction to the inflammatory site and neutrophil degranulation [17]. A_1_ARs and A_2A_ARs are also responsible for the dual role of Ado on neutrophil phagocytic activity, which is increased by former and reduced by the latter. Similarly, A_1_AR activation induces reactive oxygen species generation by neutrophils, while A_2A_ARs have an opposite effect [2]. Moreover, in vitro studies demonstrated that agonists for the A_2B_ARs and A_3_ARs are capable of blocking stimulus-induced superoxide production in wild-type but not in A_2B_AR- or A_3_AR-deficient neutrophils, thus highlighting a receptor-mediated specific effect [2]. Finally, A_3_ARs are involved in the formation and extension of filipodia-like projections in neutrophils, as shown by the selective agonist 2-chloro-N^6^-(3-iodobenzyl)-adenosine-5′-N-methyluronamide (Cl-IB-MECA), thus improving bacterial phagocytosis and chemotaxis [18]. Interestingly, Ado signaling is involved in the formation of the so-called Neutrophil Extracellular Traps (NETs), which are chromatin filaments coated with pro-inflammatory and effector molecules released by neutrophils into the extracellular space in response to inflammatory triggers [19]. NETs formation has the main role of containing pathogen spreading, but it has been also linked to autoimmune disorders, since their bioactive molecules can directly damage the extracellular matrix and amplify the immune response. In this scenario, Ado has proven its ability to reduce NETs formation and the excessive so-called “NETosis”, mostly via activation of A_2A_ARs, thus contributing to down-regulate NET pro-inflammatory activity, and to counteract the development of autoimmunity [19].

#### 1.2.2. Adaptive Immunity

Ado receptors are also expressed by B and T lymphocytes, cells involved in the adaptive or antigen-specific immunity.

Due to their role in secreting antibodies and presenting antigens, B lymphocytes are the main actors in the humoral component of the adaptive immune system, and mature to plasma cells following the contact with specific antigens. The involvement of Ado signaling in the regulation of B lymphocyte functions has been well documented, starting from the demonstration that all four ARs are expressed by murine and human B cells, together with ecto-enzymes and membrane nucleoside transporters (for review see Reference [20]). In particular, it has been observed that inactivated B cells remain under the inhibitory effect of autocrine and paracrine Ado, whereas activated B cells increase ATP synthesis and release. ATP in turn protects B cells from Ado-induced inhibition, exerts a pro-inflammatory effect on the target tissues, and stimulates IgM release [20]. On the other hand, studies on rodents showed that Ado synthesis is necessary for the development, implantation and maintenance of the plasma cell population in the bone marrow during the primary immune response. Moreover, Ado plays an important role in immunoglobulin class switching, which is a key mechanism of humoral immune response [20].

Ado also regulates the function of B regulatory cells (B_regs_), a subset of immunosuppressive B cells supporting immunological tolerance. Depending on their activation status, B_regs_ regulate both their own function and T cell activity via Ado, originating from the enzymatic degradation of ATP released in the extracellular space from activated immune cells by CD73 and CD39 enzymes, which are abundantly expressed by this cell population [21]. Under a resting condition, B_regs_ promote T lymphocyte response, while activated B_regs_ display an increased ability to produce Ado, thus inhibiting activated T cells [22].

At variance from B cells, T lymphocytes are responsible for cell-mediated immune response. T cells are activated following antigen presentation by antigen-presenting cells (APC) and are then responsible for regulating other immune cell functions by recruiting macrophages, neutrophils, eosinophils, and basophils, and by increasing their cytokine/chemokine secretion and anti-microbial activity [23]. A_2A_ARs are the most important Ado receptors in the regulation of T cells’ activation, playing an overall inhibitory effect. Previous studies summarized in Reference [2] demonstrated that A_2A_AR activation inhibits IL-4 and IFN-γ production by both naïve CD4+ T cells and Th1 and Th2 helper cells. Moreover, A_2A_AR signaling upregulates the expression of the negative co-stimulatory molecules cytotoxic T-lymphocyte antigen 4 (CTLA4) and programmed cell death 1 (PD1), and inhibits the expression of the positive co-stimulatory molecule CD-40L. In parallel, A_2A_ARs inhibit IL-2 release by polarized type 1 cytotoxic T (TC1) and TC2 CD8+ cells [2]. Finally, a recent paper demonstrated a critical role of A_2A_ARs in maintaining T helper/T regulatory (T_regs_) cells ratio, as well as the overall T/B lymphocytes ratio within the germinal centers in secondary lymphoid organs, i.e., the spleen and lymph nodes [24].

A_2A_ARs are also directly involved in the function of T_regs_, a specialized sub-lineage of T lymphocytes that modulate the immune system and maintain tolerance to self-antigens. T_regs_ play an immunosuppressive role through the inhibition of the induction and proliferation of effector T cells [25]. T_regs_ express CD39 and CD73 ectoenzymes [26], which convert extracellular ATP into Ado (see above). Ado, in turn, activates A_2A_ARs expressed on T helper cells suppressing their function and inhibits the NF-κB signaling pathway, thus reducing the release of pro-inflammatory mediators [27]. Moreover, A_2A_ARs engagement on T_regs_ induces their expansion, thereby causing additional immunosuppression with a self-reinforcing loop [28]. Recent evidence also demonstrated a competitive effect of A_2A_ARs expressed by γδ T-cells (a subtype of T cells that expresses a unique T-cell receptor, TCR, and are thought to specifically recognize lipid antigens) on Ado-mediated T_reg_ functions, since activated γδ T-cells upregulate A_2A_ARs, thus depriving T cells of local Ado and in turn inhibiting their expansion [29]. Similarly, A_2B_AR mRNA is upregulated on activated T_regs_, and endotoxin-induced T_reg_ accumulation is impaired in A_2B_AR-deficient mice [30].

In line with its role as a “retaliatory metabolite” (see above), Ado also acts as a critical immunosuppressive signal in the hypoxic microenvironment of cancer tissue, and Ado signaling can inhibit the antitumor immune response of CD8+ T cells via A_2A_ARs. This might lead to the immune escape of cancer cells which paves the way to the development of metastases [31]. Based on this, new anticancer approaches targeting Ado-mediated pathways on immune cells (e.g., anti-CD39 immunotherapy) are currently underway [32]

### 1.3. Adenosine Signaling and Autoimmunity

Autoimmune diseases are the consequence of an abnormal immune response against self-antigens. In physiological conditions, the immune response is regulated by a fine equilibrium between immunosuppressive and immunostimulatory signals that, when disturbed, leads to the development of autoimmune diseases. This cluster of pathologies share some common features, including high levels of pro-inflammatory cytokines and chemokines and massive presence of infiltrating cells such as phagocytic macrophages, neutrophils, self-reactive CD4+ T effector cells, and self-reactive CD8+ cytolytic T cells, along with a smaller amount of NK cells, mast cells, and dendritic cells. Among T effector cells, Th1, Th17, and Th9 cells mostly contribute to the pathogenesis of autoimmune diseases [33].

Among other causes, the development of autoimmune diseases has been associated with the deficiency of immunosuppressive signals. As mentioned above, ATP release during the early phases of the immune response promotes rapid inflammation by activating specific receptors, and contributes to activate T cells as well as innate immune signaling. Indeed, the role of ATP as a DAMP is also due to its strong ability to promote inflammasome activation in macrophages and dendritic cells. The accumulation of extracellular ATP characterizes the acute phase of the purinergic signaling response during inflammation, which lasts minutes to hours and is followed by a subacute phase, in which the extracellular ATP/Ado ratio declines. This phase is characterized by a reduction of ATP signaling paralleled by an increase in AR activation, aimed at containing the duration and intensity of the immune response. Extracellular Ado is normally taken up via nucleoside transporters and rapidly metabolized; however, under inflammatory conditions, the high amount of generated Ado accumulates in the extracellular space, thus exerting strong anti-inflammatory and immunosuppressive effects [34]. Accumulating evidence shows that the Ado signaling pathway mediates immune suppression by stimulating the release of anti-inflammatory cytokines (i.e., IL-10) and inhibiting the release of pro-inflammatory molecules (i.e., TNF-α and NO), thus playing an important role in maintaining the homeostasis of the immune system (see also above) [35]. When dysregulated, this potent Ado-mediated immunosuppressive machinery can contribute to the development of autoimmune disorders.

Evidence also suggests that the Ado signaling pathway is necessary for T_regs_ to exert their suppressive function. Besides Ado receptors, changes in the expression levels of CD39 and CD73 enzymes could modify ATP/Ado ratio, thus contributing to enhancing/suppressing the immune response, and finally to the development of autoimmunity (for review see Reference [36]). For example, CD39 expression was found reduced in T_regs_ from psoriasis patients [37]. As mentioned above, T_regs_ express high levels of CD39 and CD73 [26], and the generated Ado contributes to inhibiting the functions of effector T cells through A_2A_ARs. This inhibitory effect can be counteracted by effector T lymphocytes through ADA activity, which is responsible for Ado degradation [35]. Notably, not only T_regs_-generated Ado acts autocrinally on A_2A_ARs expressed by T_regs_ themselves. Receptor recruitment leads to an increase of CD39 expression, thus generating an auto-amplifying loop of immunoregulatory Ado signaling [36]. CD39 also promotes type 1 regulatory T cell (Tr1) differentiation by depleting extracellular ATP, and contributes to Tr1 suppressive activity through the generation of Ado [38]. NK and B_reg_ cells also express high levels of CD39 and CD73 and release a high amount of Ado. The former act as regulatory cells by inhibiting CD4+ T cells proliferation [39], while the latter contribute to inhibiting effector T cell functions [22].

Therefore, it is widely accepted that Ado acts as an immunosuppressive mediator that contributes to reinforcing immunological tolerance, and alterations in Ado signaling, including modification in the expression and functions of receptors and enzymes, could be linked to the development and maintenance of autoimmune disorders. Pharmacological strategies aimed at correcting these alterations are therefore potentially innovative and useful approaches to autoimmune clinical conditions.

## 2. Drugs Targeting the Adenosine System in Autoimmune Disorders: The Puzzling Cases of Methotrexate and NSAIDs

Rheumatoid arthritis (RA) is a chronic autoimmune disorder with a global prevalence of 0.24%, and females are affected twice as much as men [40]. It is characterized by a diffuse and progressive degeneration of joints due to the infiltration of white blood cells, the attack of synovium by pro-inflammatory mediators, followed by the thickening of the tissue (with the growth of the so-called *pannus*) which invades and progressively destroys joints and bones, leading to pain and disabling deformations [41]. Other organs, such as the brain and the lungs, can be less severely affected. The pathogenesis of RA has not been fully elucidated yet. It is undoubtedly an autoimmune disorder, which is triggered in a genetically predisposed individual in the presence of one or more environmental predisposing factors, including smoking, trauma, obesity, hormonal factors, irritants and pollutants, and mucosal inflammation due to specific bacterial pathogens which in turn could contribute to altering gut microbiota composition [42].

Methotrexate is a drug of choice for RA patients. It has been originally designed and developed about 80 years ago as a folate antagonist to be utilized at high doses (up to 1 g) in several types of cancers. Inhibition of folate reductase and of other enzymes involved in the synthesis of purine and pyrimidine bases blocks cells at the S phase of the cell cycle leading to cell death due to aberrant signaling pathways [43].

Starting from the 80s, methotrexate was found effective in RA at much lower doses (15–25 mg per week) than those utilized as an anti-cancer agent; interestingly, RA patients usually take daily tablets of folate to overcome possible side effects of the drug, and this does not reduce its efficacy. Thus, mechanisms other than folate antagonism should be at the basis of the efficacy of methotrexate in autoimmune disorders [44]. Actually, despite the development of new disease-modifying anti-rheumatic drugs (DMARDs), and although its mechanism of action has not been fully elucidated, methotrexate still represents a drug of choice for many RA patients after about 40 years. Several hypotheses have been raised, spanning from the generation of cytotoxic reactive oxygen species to the reduction of adhesion molecules and chemotaxis of inflammatory cells. Methotrexate mechanism of action also includes changes in the production of cytokines and inhibition of the generation of polyamines, which accumulate in the affected joints of RA patients and can be converted into molecules with a toxic effect on lymphocytes, including hydrogen peroxidase and ammonia, which have been overall referred to as “lymphotoxins” [44]. It can be envisaged that all of these pathways can somehow contribute to the beneficial effects of methotrexate in RA, but none of them likely represent the predominant one. Rather, a fundamental role seems to be played by interference with Ado-mediated signaling pathways.

First of all, peculiar pharmacokinetics leads to methotrexate accumulation within inflamed joints through the folate transporter 1 followed by the intracellular addition of several glutamate moieties, which causes the cytoplasmic entrapment of polyglutamate methotrexate [45]. The latter is thought to represent the active form of the drug, responsible for its DMARD activity, and although accumulation is a slow process it can persist within inflamed joints far beyond the rapid breakdown of methotrexate plasma concentrations [45].

Among the various enzymes involved in the synthesis and metabolism of purine and pyrimidine bases, methotrexate is known to inhibit the 5-aminoimidazole-4-carboxamide ribonucleotide (AICAR) transformylase (ATIC) enzyme which converts AICAR to formyl AICAR (FAICAR), an intermediate in the synthesis of purines, thus leading to the intracellular increase of AICAR which in turn is a potent inhibitor of ADA [46]. The reduction in Ado catabolism to inosine increases intracellular Ado concentrations, leading to a subsequent increase in the extracellular release of Ado through bidirectional equilibrative nucleoside transporter 1 (ENT1); thus, by intracellularly interfering with Ado degradation, methotrexate can increase its extracellular availability. This is likely to increase the recruitment and activation of Ado receptors on immune cells, leading to an overall immunomodulatory activity [46].

Several in vitro and in vivo studies with the use of AR antagonists or KO mice for specific AR subtypes have highlighted a prominent role for A_2A_ARs in the DMARD activity of methotrexate [44]. In line with these data, it has been observed that naturally-occurring methylxanthines (i.e., caffeine and theophylline), which are known to act as AR antagonists, can significantly reduce methotrexate efficacy in rats [47]. In a small cohort of patients, the presence of 4 A_2A_AR Single Nucleotide Polymorphisms (SNPs) did not modify patients’ response to methotrexate, but has been correlated to a reduced relative risk to develop side effects [48]. Although the functional outcomes of these SNIPs are not yet understood, these data confirm the involvement of A_2A_ARs in the pharmacological activity of methotrexate in RA, including the development of side effects. Along with A_2A_ARs, A_3_ARs are the most expressed Ado receptors in synoviocytes, thus suggesting their role in RA pathogenesis [49] (see also Section 3), but also their possible exploitation as drug targets. Interestingly, in line with this, a higher basal level of expression of A_3_ARs in the whole peripheral blood of RA patients at the beginning of methotrexate monotherapy was found in good or moderate responders to the drug with respect to non-responder patients [50]. More recently, certain polymorphisms in the *ATIC* gene have been found to be associated either to the positive outcome of methotrexate treatment or the insurgence of side effects [51], and a low level of expression of CD39 on T_regs_ was observed in non-responders to the drug [52]. Taken together, these clinical data confirm the central role of Ado signaling pathways in regulating the efficacy of methotrexate in RA.

Ado-mediated signaling is also involved in the effects of other drugs utilized in RA. The administration of high daily doses of non-steroidal anti-inflammatory drugs (NSAIDs), including acetyl salicylic acid, has been long considered one therapeutic option for RA patients, based on their known ability to inhibit pro-inflammatory cyclooxygenases. This regimen has now been abandoned, but it has allowed us to identify multiple pathways activated by the drug chemical precursor, salicylate, which include an indirect elevation of extracellular Ado concentrations [53]. A similar indirect influence on the Ado system has been demonstrated for high doses of other NSAIDs (i.e., sulindac) and for sulfasalazine, a prodrug of 5-amino-salicylic acid acting as a DMARD [53,54].

Overall, these data allow depicting an Ado-based scenario for the clinical efficacy of methotrexate and sulfasalazine as DMARDs and for high doses of NSAIDs as adjuvant anti-inflammatory agents in RA, and pave the way for new therapeutic approaches aimed at directly targeting specific Ado receptors to further improve pharmacological responses while reducing side effects related to the generalized tissue increases in Ado concentrations induced by methotrexate.

## 3. Towards New Ado-Based Immunomodulatory Approaches: Preclinical and Clinical Studies

### 3.1. The Beneficial Role of A_1_ARs in Autoimmunity

Since it has been one of the first members of the AR family to be discovered, the A_1_AR subtype has been extensively characterized. It is activated by physiological Ado concentrations, and couples to IP3 generation but also to K^+^ channels especially in the central nervous system (CNS) and in the heart. Thus, it is responsible for the various inhibitory effects of Ado, including its action as an antiepileptic, sleep inducer, and bradycardic agent [49]. Its blockade is at the bases of most of the excitatory effects induced by naturally-occurring methylxanthine compounds, i.e., caffeine, theophylline, and theobromine.

The overall role of A_1_ARs expressed by the immune system is not univocal, as both pro- and anti-inflammatory effects have been described, depending on both the cell type and the pathological condition [55]. However, when speaking of autoimmune disorders, evidence suggests a protective role for A_1_AR-mediated signaling.

This was first reported in multiple sclerosis (MS), a neuroinflammatory autoimmune disease of the CNS in which the immune system attacks the myelin sheath enwrapping nerves, and in experimental autoimmune encephalomyelitis (EAE), the animal model of MS [56].

A_1_AR activation regulates the levels of pro-inflammatory cytokine TNF-α and of IL-6, a cytokine with both pro- and anti-inflammatory effect, in relapsing-remitting MS (RR-MS) patients. Authors observed that serum levels of TNF-α are significantly higher and those of Ado are significantly lower in RR-MS patients with respect to control subjects. Moreover, activation of A_1_ARs expressed by peripheral blood mononuclear cells (PBMCs) significantly reduces TNF-α but not IL-6 levels in control subjects, with the opposite occurring in RR-MS patients, in which reduced levels of A_1_ARs were also observed [57]. These findings were later confirmed by a study showing that A_1_AR mRNA levels are reduced in the brain and in PBMCs of MS patients compared to patients with other neurological diseases and controls. The authors also detected increased TNF-α expression in the brain of MS patients, together with decreased A_1_AR levels on monocytes. In addition, the A_1_AR-specific antagonist 8-cyclopentyl-1,3-dipropylxanthine (DPCPX) reduces the ability of adenosine analogues to inhibit TNF-α production by stimulated monocytic cell lines [58]. Studies on rodents showed that A_1_ARs regulate the severity of motor score and neuropathological outcomes in EAE, such as demyelination and axonal damage, and receptor activation reduces inflammation in the CNS [58]. 

It is well known that the pathogenesis of MS involves the release of pro-inflammatory mediators by activated microglia and macrophages, which contributes to myelin and white matter damage. In MS patients, the reduced expression of A_1_ARs likely contributes to disease progression by decreasing the ability of Ado to regulate the inflammatory response in the brain [58]. Furthermore, another study demonstrated that A_1_ARs on microglial cells are downregulated after EAE induction, and administration of glucocorticoids, commonly used in MS treatment, is able to restore receptor expression and, likely, its anti-inflammatory effects [59].

In vivo studies showed that, compared to wild-type littermates, A_1_AR null (A_1_AR^−/−^) mice develop a more severe form of EAE, characterized by increased demyelination, axonal injury and enhanced activation of microglia/macrophages. In addition, spinal cords from A_1_AR^−/−^ EAE mice display increased pro-inflammatory gene expression, whereas anti-inflammatory genes are reduced. Macrophages from A_1_AR^−/−^ mice also show increased expression of pro-inflammatory genes, IL-1β, and of the neurotoxic mediator matrix metalloproteinase-12 (MMP12), and A_1_AR^−/−^ macrophage-derived soluble factors cause oligodendrocyte cytotoxicity compared to wild-type control cells. Notably, MMP12 mRNA expression is also increased in the brain of MS patients [59].

It has been reported that chronic caffeine treatment reduces EAE symptoms, cell infiltration, demyelination, and pro-inflammatory cytokine secretion, probably due to rebound overexpression of A_1_ARs and downregulation of IFN-γ mRNA [60]. In agreement with this hypothesis, it was previously shown that in vitro exposure to caffeine of human monocytoid cells increases A_1_AR expression and decreases pro-inflammatory cytokine secretion. The same was shown on microglia, resulting in a reduction of EAE severity [59]. Subsequently, it was reported that caffeine administration during the whole course of EAE (0–20 days post-immunization, DPI, with myelin oligodendrocyte glycoprotein, MOG) reduces CNS demyelination and relieves inflammatory injury by reducing pro-inflammatory and increasing anti-inflammatory cytokines. Similar beneficial effects were observed if caffeine is administered during the effector (10–20 DPI) but not during the induction (0–10 DPI) phase of EAE. Caffeine-induced neuroprotection is associated with the reversal of A_1_AR mRNA down-regulation [61].

Additional studies showed that beta-arrestin-1 expression is increased in the brain of MS patients, while A_1_AR expression is reduced, and this inverse relationship was also observed in cultured monocytoid cells, and in the spinal cord of EAE mice. In monocytoid cells, beta-arrestin-1 overexpression results in a down-regulation of A_1_ARs with internalization of the surface receptor through a physical interaction between the two proteins, which was regulated by pro-inflammatory cytokines. Moreover, EAE-induced neuroinflammation and neurobehavioral deficits in mice are inhibited by glucocorticoid treatments, in parallel with reduced beta-arrestin-1 and enhanced A_1_AR expression, thus confirming the fundamental role played by A_1_ARs during neuroinflammation [62].

A recent paper provides further hints on the mechanisms driving the Ado immunosuppressive function in EAE. Authors demonstrated that activation of A_1_ARs on astrocytes leads to decreased pro-inflammatory cytokine and chemokine production, and attenuates the stimulating effect of astrocytes on effector T cells. Finally, in vivo experiments confirmed that local administration of the A_1_AR agonist 2-Chloro-N^6^-cyclopentyladenosine (CCPA) exerts a protective effect on EAE in wild-type but not A_1_AR^−/−^ mice [63]. Interestingly, a pro-inflammatory effect of CD73 downregulation in EAE astrocytes was recently demonstrated. In this study, CCPA showed the same effect as Ado on CD73^−/−^ astrocytes, while other selective AR agonists did not [64].

A_1_ARs seem to play a protective role also in type 1 diabetes (T1D), an autoimmune disease characterized by destruction of the insulin-producing pancreatic β-cells by the immune system. Pancreatic α-cells also play a key role in the regulation of glycaemia, since glucagon secretion from these cells triggers hepatic glucose release and, ultimately, prevents hypoglycemia [65]. No definitive cure is available, with current treatments only aimed at controlling blood sugar levels by insulin replacement therapies, in order to prevent vascular, neurological, and other tissue complications. In this scenario, a study reported that A_1_AR expression in pancreatic α-cells is reduced in both diabetic NOD mice and T1D patients. Since it has been demonstrated that A_1_ARs also act as glucagon inhibitors, their downregulation in α-cells possibly contributes to glycaemia dysregulation and to the development of the pathology [66].

There is growing evidence that defects in the Ado signaling pathway contribute to the loss of immunotolerance also in autoimmune liver disorders. Studies in a murine model of experimental cholestasis reported a pathogenic role for A_1_AR signaling in mediating liver injury, as the lack of A_1_ARs limits the efflux of toxic biliary constituents through the biliary excretory route [67].

### 3.2. The Immunomodulatory Role of A_2A_ARs: Can Its Derailment Play a Role in Autoimmune Disorders?

Similarly to the A_1_AR subtype, A_2A_ARs are activated by low physiological Ado concentrations and activate adenylyl cyclase through G_s_. They are widely distributed in the periphery, especially on platelets and vessels (including coronary vessels), where they inhibit platelet aggregation and promote vasodilation, and in the CNS. In the *corpus striatum,* their peculiar localization on neurons also expressing the D2 dopamine receptors, which inhibits adenylyl cyclase, is at the bases of the functional antagonism between Ado and dopamine on the control of voluntary movements [55]. The functional equilibrium between these two systems is pathologically altered in Parkinson’s disease, where overactivation of A_2A_ARs is responsible for many of the clinical manifestations of the pathology. For this reason, the first A_2A_AR antagonist Istradefylline has been approved as an adjunct therapy for this disease [68].

A_2A_ARs are also widely expressed in the immune system, and their immunomodulatory role has been known for many years, with evidence indicating that the massive accumulation of Ado at inflamed or injured sites acts as “stop” signal that senses a potentially dangerous and excessive immune response and blocks its pathological spreading. This “retaliatory” activity of Ado is based on the activation of A_2A_ARs (along with A_2B_ARs, see below) on immune cells [69]. Later on, a potential role for this receptor subtype as a pharmacological target in autoimmunity has progressively emerged.

As mentioned above, both A_2A_ARs and A_3_ARs are highly expressed by human synoviocytes, the cell population mostly involved in RA [41], and both receptors promote an anti-inflammatory response, despite their different sensitivity to the natural agonist Ado and the coupling to different second messengers (i.e., stimulation of cAMP and IP3 formation, respectively) [70]. This is in line with the overall effects of A_2A_AR activation on immune cells in RA, showing anti-inflammatory and immunomodulatory activity. A_2A_ARs inhibit TNF-α production by macrophages and upregulate IL-10 expression. Additionally, they promote overall anti-inflammatory effects on mature dendritic cells, neutrophils, and lymphocytes (see above) [41,46].

These functional data are fully confirmed by pre-clinical studies in rodent models of RA exposed to selective A_2A_AR agonists. A significant improvement of the rheumatic status of rats have been observed with the administration of 2-p-(2-carboxyethyl)phenethylamino-5′-*N*-ethyl carboxamidoadenosine (CGS21680), one of the first selective A_2A_AR agonists ever, possibly through the modulation of the IL-10 pathway [41,71]. This anti-inflammatory activity has been confirmed with a very recent non-purinergic A_2A_AR selective agonist, 3,4-dimethoxyphenyl-N-methyl-benzoylhydrazide (LASSBio-1359), which has been reported to modulate the production and release of cytokine, thus ameliorating RA-related inflammatory pain [72].

Based on the above-mentioned data, A_2A_AR (and A_3_AR, see below) agonists likely represent an interesting future possibility for the development of new anti-RA DMARDs. This is further confirmed by an interesting upregulation in the expression of both receptor subtypes on lymphocytes and neutrophils from RA patients, which inversely correlates with disease activity score (i.e., the higher the receptor expression, the lower the severity of the disease) [71,73]. The activity of both receptors is associated with the suppression of pro-inflammatory cytokine and MMP release, i.e., to a most favorable disease outcome [71], further highlighting their clinically favorable immunomodulatory effects.

A similar immunomodulatory role for A_2A_ARs has been demonstrated in inflammatory bowel disease (IBD), a group of autoimmune gastrointestinal disorders including Crohn’s disease and ulcerative colitis, whose development is characterized by visceral hypersensitivity, where A_2A_AR agonists reduce the release of pro-inflammatory cytokines, increase T_reg_ cell activity, and reduce leukocyte infiltration [74].

A protective effect for A_2A_ARs is also hypothesized in Systemic Lupus Erythematosus (SLE), a chronic, relapsing-remitting, multiorgan autoimmune disease whose clinical manifestations can affect the musculoskeletal, renal, respiratory, cardiovascular, and central nervous systems, as well as the blood and the skin. Immune cells in SLE secrete increased amounts of cytokines, mostly type I interferons, and other soluble pro-inflammatory mediators, which cause inflammation and the destruction of end-organs [75]. Higher expression of A_2A_ARs has been found on T cells from SLE patients with respect to healthy subjects, which inversely correlated with disease severity [76]. Additionally, CGS21680 was more effective in dampening the production of T cell pro-inflammatory cytokines in SLE patients than in control subjects. The authors have therefore hypothesized that A_2A_AR upregulation represents an attempt to counteract the inflammatory milieu in SLE [76]. This hypothesis is further confirmed by a deficient CD39 and CD73 activity in lymphocytes from SLE patients, which in the end would lead to a reduced Ado-mediated immunosuppression over the course of the pathology which could be partially counteracted by A_2A_AR upregulation [75].

A more complex scenario emerges from studies in MS and in its animal model, EAE. In line with its above-mentioned immunomodulatory role, the genetic deletion of A_2A_ARs generates a more severe and aggressive form of EAE with pronounced demyelination and neurological deficits [77]. Animal data were indirectly confirmed by data on human MS lymphocytes showing upregulation of A_2A_ARs, which promote an anti-inflammatory effect when stimulated [78]. Conversely, a protective effect has been demonstrated in wild-type animals for the administration of the A_2A_AR antagonist 7-(2-phenylethyl)-5-amino-2-(2-furyl)-pyrazolo-[4,3-e]-1,2,4-triazolo[1,5-c]pyrimidine (SCH58261) with a reduction of CNS neuroinflammation, macrophage infiltration, and microglia activation and overall amelioration of the pattern of demyelination and associated neurological deficits [79]. The most likely explanation for this paradoxical outcome resides in the time window for the administration of the drug, which is effective only when administered from day 11 to day 28 post-immunization roughly corresponding to a putative time window for the treatment of MS patient, but not when administered immediately post immunization [79]. These data highlight a differential role played by A_2A_ARs expressed by either immune or CNS cells during the course of the pathology, and additional paradoxical effects have been observed when dissecting the role of this receptor subtype within the complex scenario leading to the development of EAE along with the obvious limitations due to the use of animal models (as brilliantly reviewed in Reference [80]).

An unexpected opposite action played by A_2A_ARs has been also demonstrated in mouse autoimmune uveitis (EAU), an inflammatory process of the uveal components of the eye caused by an autoimmune response to self-antigens or by an innate inflammatory reaction secondary to an external stimulus. At variance from several other autoimmune disorders (see also below), in this specific pathological setting, both A_2A_ARs and A_2B_ARs promote disease progression, mostly through a boost in the activation of γδ T cells, which express increased levels of A_2A_ARs in parallel with reduced expression of CD73. Consistently, the exposure to A_2A_AR antagonist rebalances the ratio between enhancing and inhibiting functions of γδ T cells, thus opening the question of whether a derailment of the A_2A_AR-mediated immunomodulatory action of Ado could contribute to autoimmunity at least in some specific pathological settings and suggesting that the blockade of Ado activity could represent an interesting adjuvant approach to AU [81]. It is worth mentioning that a spontaneous recovery from EAU was observed in mice 75–90 days post-immunization with no further relapse. The main drivers of recovery are APC expressing high levels of A_2A_ARs and the melanocortin 5 receptor, whose activation leads to increased CD73 and CD39 expression, with an increase of extracellular Ado concentrations which in turn promotes T_reg_ activation and resolution of uveitis [82]. Based on these conflicting results, caution should be used when planning an A_2A_ARs-based therapy for uveitis since whether an agonist or an antagonist will provide the better outcome might depend upon the stage of the disease.

### 3.3. Interesting Hints from the Long Considered Elusive Member of AR Family: The A_2B_AR

Up to mid 90s, the role and functions of A_2B_ARs were elusive. It was cloned and its low affinity for the natural ligand Ado was known, suggesting its prominent role under pathological conditions, i.e., when extracellular Ado concentration rises several-fold. It was only with the discovery of the potent A_2B_AR-mediated bronchoconstriction that this receptor has become one of the most promising targets for Ado-based therapy, especially in asthma and chronic obstructive pulmonary disease (COPD) [83]. Later, its role in modulating immune functions has emerged along with the possible involvement in autoimmunity.

The role of A_2B_ARs in the pathogenesis of MS is less clear with respect to other members of AR family, but seems overall detrimental. As for the A_2A_ARs and A_3_ARs, A_2B_ARs are upregulated in both peripheral blood leukocytes of MS patients and peripheral lymphoid tissues of EAE mice. In addition, A_2B_AR antagonists 3-ethyl-3,9-dihydro-1-propyl-8-[1-[[3-(trifluoromethyl)phenyl]methyl]-1*H*-pyrazol-4-yl]-1*H*-purine-2,6-dione (CVT-6883) and 8-[4-[((4-Cyanophenyl)-carbamoylmethyl) oxy]phenyl]-1,3-di(n-propyl) xanthine (MRS1754) alleviate the clinical symptoms of EAE and protect the CNS from immune damage, and A_2B_AR-knockout mice develop less severe EAE. In particular, CVT-6883 administration or genetic deletion of A_2B_ARs inhibit Th17 cell differentiation by blocking IL-6 production from APC including dendritic cells, in which this receptor subtype is upregulated during the development of the pathology. The PLCβ/protein kinase C and p38 MAPK pathways were found to be involved in the A_2B_ARs-mediated IL-6 production [84]. Very recently, a research group investigated the effect of mesenchymal stem cell transplantation in mice at the onset of EAE, and observed that the treatment improves neurobehavioral outcomes, reduces inflammatory cell infiltration, IgG leakage, and demyelination in the spinal cord, with a significant downregulation of aquaporin-4 (AQP4) and A_2B_AR expression. Moreover, mesenchymal stem cells-conditioned medium (MCM) reduces the expression of inflammatory cytokines, AQP4 and A_2B_ARs in lipopolysaccharide (LPS)-activated astrocytes, effects that are reversed by the selective A_2B_AR agonist 2-[[6-Amino-3,5-dicyano-4-[4-(cyclopropylmethoxy)phenyl]-2-pyridinyl]thio]-acetamide (BAY-60-6583). Furthermore, the effects of BAY-60-6583 are counteracted by p38 MAPK inhibitor 4-(4-Fluorophenyl)-2-(4-methylsulfinylphenyl)-5-(4-pyridyl)-1*H*-imidazole (SB203580), thus demonstrating that mesenchymal stem cells prevent EAE-induced damage by downregulating AQP4, possibly through the inhibition of the A_2B_/p38 MAPK signaling pathway [85].

As mentioned above, A_2B_ARs exert stimulatory effects on Th17 autoreactive and γδ T cells. Consistently, administration of a selective A_2B_ARs agonist to C57BL/6 mice greatly enhances the development of EAU with increased Th17, but not Th1, responses. A protective effect was instead observed when treating mice with an A_2B_AR antagonist. The authors further demonstrated that agonist-induced enhancement of the Th17 response is significantly lower in TCR-δ^−/−^ mice, and that transfer of γδ T cells into TCR-δ^−/−^ mice partially restores sensitivity to A_2B_AR agonist. Moreover, dendritic cells from A_2B_AR agonist-treated mice show a significantly increased ability to activate γδ T cells and Th17 autoreactive T cells [86]. As a confirmation, A_2B_AR activation switches the differentiation of bone marrow cells to a dendritic cell subset that promotes Th17 response [87].

Bazzichi and coworkers also investigated protein expression and function of A_2A_ARs and A_2B_ARs in neutrophils of patients affected by systemic sclerosis (SSc), an autoimmune rheumatic disease characterized by widespread fibrosis in the skin and internal organs and by injuries to small arteries. While no significant changes in A_2A_AR binding parameters or expression levels are detected, a significant decrease in the maximum density of A_2B_AR binding sites occurs in SSc neutrophils, in parallel with decreased A_2B_ARs-mediated activation of adenylyl cyclase activity [88]. These data were recently confirmed in a murine model of SSc, where the administration of the orally active A_2B_AR antagonist 3-Ethyl-3,9-dihydro-1-propyl-8-[1-[[3-(trifluoromethyl)phenyl]methyl]-1*H*-pyrazol-4-yl]-1*H*-purine-2,6-dione (GS-6201) reduces the production of pro-fibrotic mediators in the skin, attenuates dermal fibrosis, and reduces the number of arginase-expressing macrophages and myofibroblasts and the levels of extracellular matrix proteins, suggesting A_2B_ARs as potential pro-fibrotic regulators [89].

There is also evidence to suggest a role for A_2B_ARs in T1D but, at variance from MS, EAU and SSc (see above), it seems to be overall protective. Studies on murine models of streptozotocin-induced diabetes suggest that the inhibition of pro-inflammatory cytokine release is predominantly mediated by A_2B_AR activation [90]. Moreover, high levels of CD39, in association with high A_2A_AR and A_2B_AR expression in T helper cells, have been found to be protective in the same experimental model [91].

A number of studies have demonstrated the involvement of A_2B_ARs signaling in the immunopathogenesis of IBD. The current knowledge has led to the conclusion that A_2B_ARs play a role in intestinal inflammation, although it is not fully clear whether it is pro- or anti-inflammatory, with most evidence pointing to the latter [92]. The first reports showed that A_2B_ARs activation on intestinal epithelial cells increases IL-6 production, resulting in increased neutrophil activation, and upregulation of A_2B_AR expression has been observed in epithelial cells from mouse or human colitis [93]. The administration of the selective A_2B_AR antagonist 1-cyclopropyl-3-propyl-8-(6-(*N*-nicotinoyl-*N*-ethylamino)-3-pyridyl)xanthine (ATL-801) to a mouse model of colitis markedly decreases IL-6 secretion and neutrophil infiltration, and reduces the extent of mucosal damage, thus ameliorating the disease course [94]. The pro-inflammatory role of A_2B_ARs was confirmed in A_2B_AR-knockout mice, in which colitis results to be attenuated [95].

Data also show the participation of A_2B_ARs to the modulation of intestinal peristaltic activity, since the administration of the A_2B_AR antagonist MRS1754 enhances both electrically-evoked and carbachol-induced cholinergic contractions in normal rat colon, but is less effective in inflamed tissues. Conversely, the non-selective A_2B_AR agonist 5′-(*N*-Ethylcarboxamido)adenosine (NECA) decreases colonic cholinergic motility, with increased efficacy in inflamed tissue [96]. In the gastro-intestinal tract, A_2B_ARs are mainly expressed by epithelial cells of the colon and by enteric neurons, while it is still a matter of debate whether they could stimulate mast cell activity. It has been reported that somatic and visceral pain can be blocked by the inhibition of A_2B_ARs, but the precise role played by the receptors in the pathophysiology of intestinal inflammation is still unclear [92]. For example, global A_2B_AR deletion is protective against inflammatory damage induced by dextran-sulfate sodium (DSS), 2,4,6-trinitrobenzene sulfonic acid (TNBS), *Salmonella typhimurium*, and in IL-8-induced colitis [95]. However, recent studies on mice bearing A_2B_AR conditional deletion on vascular endothelial or intestinal epithelial cells attribute the ability to reduce colonic inflammation only to A_2B_ARs expressed by epithelial cells [97].

Finally, patients affected by primary biliary cholangitis, an autoimmune liver disorder, exhibit dramatic phenotypic alterations in CD8+ T_regs_, as reflected by lower CD39 expression that correlates with lower responsiveness to IL-10 [98]. A_2B_AR activation in mouse cholangiocytes promotes IL-6 release via cAMP and Ca^2+^ signaling, thus favoring cholangiocytes survival during biliary cirrhosis [99].

### 3.4. A_3_AR Agonists: Clinical Trials Are already on Their Way to Find Novel Approaches to Autoimmune Disorders

The A_3_AR subtype was the only AR discovered by cloning techniques. It has the unique feature of being virtually insensitive (at least in rodents) to xanthine derivatives, which conversely act as antagonists at the other three ARs [100]. Additionally, it shows the highest variability in sequence homology and in sensitivity to agonists and antagonists among different species, making the translation of data from animal models to the clinics more complicated than other members of the AR family [101]. Research on this unknown receptor was boosted in the early 90s by the synthesis by Prof. Ken Jacobson and his group at NIH of the first selective A_3_AR agonists, N^6^-(3-iodobenzyl)adenosine-5′-*N*-methyluronamide (IB-MECA) and its chloro derivative Cl-IB-MECA, along with a huge number of antagonists [101]. In the following decades, several groups have contributed to dissecting the multiple physiopathological roles of this receptor (reviewed in Reference [55]), and to pointing out that, together with the A_2B_AR subtype (see above), it is activated by high Ado concentrations. Its prominent role under pathological conditions could be therefore easily foreseen.

A_3_ARs are expressed virtually by all immune cells and exert a dual role in inflammation with a pro-inflammatory effect in the lung (where they increase histamine release and eosinophil and macrophage chemotaxis) and an anti-inflammatory action against LPS-induced cytokine release [102].

Conversely, when talking about autoimmune diseases, A_3_ARs share several characteristic features with A_2A_ARs with an overall immunomodulatory effect. For example, both receptor subtypes are upregulated on PBMCs of RA patients [73], and are expressed in human synoviocytes where their activation dampens inflammation through the recruitment of the phosphoinositide 3-kinase (PI3K) signaling pathway [70]. Inhibition of TNF-α release from synoviocytes, spleen- and lymph node-derived cells was demonstrated in animal models of the pathology [103]. The upregulation of A_3_ARs in a pathological setting with respect to control condition has proven to be fundamental for the possible exploitation of agonists as anti-rheumatic drugs, since it renders patients’ cells more susceptible to drug actions (see below).

IB-MECA is currently developed as an orally bioavailable pharmacological agent in autoimmune disorders with the name of Piclidenoson (CF-101) by Can-Fite BioPharma, Israel. In pre-clinical studies in rodents exposed to collagen- or adjuvant-induced arthritis, the combined therapy of methotrexate and CF 101 yielded interesting synergic anti-rheumatic activity. In fact, methotrexate induces the upregulation of A_3_ARs on immune cells, which in turn facilitates the inhibitory effect of CF101 on NF-κB pathway and, consequently, on the release of pro-inflammatory mediators [104]. Based on pre-clinical encouraging data and on the excellent safety and tolerability profile which has emerged from Phase I clinical studies, CF 101 underwent successful Phase IIa and IIb clinical studies as a standalone drug for use in humans, and significant anti-inflammatory actions have been demonstrated with no side effects. Interestingly, authors have also shown that the levels of expression of A_3_ARs in PBMCs from patients before starting the therapy positively correlate with the therapeutic efficacy of the treatment, suggesting that peripheral A_3_ARs could be also exploited as predictive markers of patient’s response to the drug [103].

Another possible approach is represented by the use of allosteric modulators acting on the receptor, which bear the potential advantage of favoring receptor activation only in the presence of the endogenous ligand, i.e., at sites where Ado concentrations reach typical pathologically high levels. In this respect, the imidazoquinoline compound 2-cyclohexyl-*N*-(3,4-dichloro phenyl)-3H-imidazo[4,5-c]quinolin-4-amine (LUF6000) has demonstrated an anti-inflammatory effect in rodent models of arthritis and of inflammatory liver disease, suggesting its potential exploitation in humans as well [101].

Psoriasis is another common autoimmune disorder characterized by excessive cell proliferation, especially in the skin with the formation of plaques, and immune-mediated inflammation. Plaque psoriasis clinically manifests with erythematous, asymmetric and thick plaques, mostly affecting the arms, trunk, and scalp. A smaller percentage of patients shows additional clinical forms of the disease, including erythrodermic, pustular, guttate, inverse, and palmoplantar psoriasis [105]. An increased risk of cardiovascular diseases has been also described. Active lesions are accompanied by pruritus, scaling, and pain. Additionally, social rejection along with reduced self-confidence and well-being are often described. Thus, psoriasis bears a significant personal and social burden and, despite the current availability of several oral and topic treatments, many patients have not found a real relief yet. This is mostly due to side effects, the high cost of the therapy, and poor patient compliance due to the route of administration [105]. More than 40 genetic mutations have been correlated with psoriasis, and it is now currently recognized that the pathology becomes manifested when a genetically predisposed individual is exposed to environmental triggers (e.g., stress, infection, trauma, or even some medications). Based on current knowledge, activated dendritic cells in the dermis act as triggers for the pathology through the increased production of IL-23, which in turn promotes IL-17 production by dysregulated CD4+ T cells (Th17 cells), innate lymphoid cells, and γδ T cells. Pathological T cells migrate to the epidermis and modify keratinocytes functions leading to the appearance of the classical psoriasis lesions [105]. Based on the key role played by A_3_ARs in controlling immune cell function and on the positive results in RA (see above), CF101 has been proposed as an innovative anti-psoriasis treatment. Results from Phase II clinical trials have been so encouraging (with 35.3% of patients reaching an amelioration of the Psoriasis Area and Severity Index, PASI, of 50%, and two additional subjects of 73% and 100%), and side effects so limited (with an incidence of 17.6% in the group treated with the most effective dose of the drug, i.e., 2 mg, compared to 21.1% in the placebo group), that the drug has entered a currently ongoing Phase III clinical study [105].

Closely related to psoriasis, psoriatic arthritis is an inflammatory musculoskeletal disease that affects approximately one-third of patients who already have psoriasis, and which can present as oligoarthritis, enthesitis, and dactylitis, as well as psoriatic nail disease [105]. In recent years, it has been demonstrated that TNF-α represents one crucial element in the development of psoriatic arthritis, and both direct and receptor TNF-α inhibitors are currently utilized as well-tolerated pharmacological treatments [106]. Several drugs targeting different cytokine pathways are also available (reviewed in Reference [106]), with a general satisfactory clinical response achieved in the majority of patients. However, treatment failure with these drugs can represent a relevant clinical problem and other targets are currently under study to develop innovative and effective treatments. The above-mentioned data from clinical studies with CF101 in RA and psoriasis and the known mechanisms of action recruited by A_3_AR activation have suggested a possible use of CF101 in psoriatic arthritis as well. No direct data are currently available, but the strong rationale makes this hypothesis extremely promising [106].

Finally, as mentioned in the previous section, expression of A_3_ARs is upregulated in the spleen and lymph nodes of mice subjected to EAE [84]. Our research group has recently demonstrated an upregulation of A_3_ARs in the brainstem of rats exposed to the pathology starting from the onset of the disease, in parallel with the development of reactive gliosis and with the insurgence of trigeminal sensitization and orofacial allodynia [107]. Although data on the contribution of A_3_ARs expressed by immune cells to the development of motor signs of EAE are not currently available, these data suggest that, based on its known role in pain pathways [108], this receptor subtype might represent an interesting pharmacological target for MS-associated pain syndromes which are often poorly controlled by available painkillers.

## 4. Conclusions

Although not exhaustive due to the increasing number of published papers, this review article highlights the fundamental role played by Ado, acting through its 4 G protein-coupled receptors, in the modulation of immune cell function both in physiological and in pathological conditions, such as autoimmune disorders, as summarized in Table 1.

Among the vast number of available and sometimes contradictory evidence, the most promising results are currently related to the A_3_AR subtype, possibly due to two fundamental issues: the characteristic upregulation of this receptor subtype in autoimmune pathologies, which allows its prevalent activation in affected tissues only, and the fundamental contribution of medicinal chemists who have made selective agonists and antagonists available for pre-clinical and clinical studies. Additionally, A_3_ARs could also be exploited as biomarkers of therapeutic efficacy, thus paving the way for personalized patient-centered therapy. The progressively more detailed understanding of the dysregulated pathways leading to autoimmune disorders and of the mechanisms of action of Ado-based drugs will hopefully allow us to extend currently available therapeutic strategies to additional autoimmune disorders.

## Figures and Tables

**Table 1:** Overview of the involvement of ARs in various autoimmune disorders.

Receptor	Autoimmune Disease	Hypothesized Role	References
A_1_	Multiple sclerosis	Protective role	[56]
	Type 1 diabetes	Reduced expression in pancreatic α-cells contributes to the pathology	[66]
	Autoimmune liver disorders	Lack of receptors limits the efflux of toxic biliary constituents	[67]
A_2A_	Reumathoid arthritis	Anti-inflammatory activity	[46]
	Inflammatory bowel disease	Anti-inflammatory activity	[74]
	Multiple sclerosis/EAE	Opposite conflicting results	[77,79]
	Experimental autoimmune uveitis	Activation promotes disease progression	[81]
	Systemic Lupus Erythematosus	Anti-inflammatory activity	[75,76]
A_2B_	Multiple sclerosis	Pro-inflammatory actions	[84]
	Experimental autoimmune uveitis	Pro-inflammatory actions	[86]
	Systemic sclerosis	Pro-fibrotic regulator in vivo	[89]
	Type 1 diabetes	Inhibition of pro-inflammatory cytokine release	[90]
	Inflammatory bowel disease	Pro-inflammatory actions	[92]
	Autoimmune liver disorders	Promotion of IL-6 release	[99]
A_3_	Reumathoid arthritis	Dampening of immune response (Phase II clinical trials with selective agonists)	[103]
	Psoriasis	Dampening of immune response (Phase III clinical trials with selective agonists)	[105]
